# Supplementary material for: Allele-Specific CRISPR/Cas9 Correction of a Heterozygous DNM2 Mutation Rescues Centronuclear Myopathy Cell Phenotypes
Source: Mol Ther Nucleic Acids. 2019 Feb 27;16:246–56. doi: 10.1016/j.omtn.2019.02.019 (PMC6439232; doi:10.1016/j.omtn.2019.02.019)
Supplement: Document S1. Figures S1–S3 [file mmc1.pdf]

OMTN, Volume *16*

Supplemental Information

Allele-Specific CRISPR/Cas9 Correction of a Heterozygous *DNM2* Mutation Rescues Centronuclear Myopathy Cell Phenotypes

Aymen Rabai, Léa Reisser, Bernardo Reina-San-Martin, Kamel Mamchaoui, Belinda S. Cowling, Anne-Sophie Nicot, and Jocelyn Laporte

**WT allele**	CTGAGTTCCTACCCCCGGCTGCGAGAGGAGACCGAGCGAATTGTCACCACCTACATCAGGGAGCGAGAAGGGAGAACCAAGGACCAG
**Mutated allele**	CTGAGTTCCTACCCCCGGCTGCGAGAGGAGACCGAGCGAATTGTCACCACCTACATCTGGGAGCGAGAAGGGAGAACCAAGGACCAG
**KI1-NHEJ1**	CTGAGTTCCTACCCCCGGCTGCGAGAGGAGACCGAGCGAATTGTCACCACCTAC--TCTGGGAGCGAGAAGGGAGAACCAAGGACCAG
**KI1-NHEJ2**	CTGAGTTCCTACCCCCGGCTGCGAGAGGAGACCGAGCGAATTGTCACCAC----------TCTGGGAGCGAGAAGGGAGAACCAAGGACCAG
**KI1-NHEJ3**	CTGAGTTCCTACCCCCGGCTGCGAGAGGAGACCGAGCGAATTGTCACCACCTAC--TCTGGGAGCGAGAAGGGAGAACCAAGGACCAG
**KI1-NHEJ4**	CTGAGTTCCTACCCCCGGCTGCGAGAGGAGACCGAGCGAATTGTCACCA------------TCTGGGAGCGAGAAGGGAGAACCAAGGACCAG
**KI1-NHEJ5**	CTGAGTTCCTACCCCCGGCTGCGAGAGGAGACCGAGCGAATTGTCACCACCTACATTTCTGGGAGCGAGAAGGGAGAACCAAGGACCAG
**KI1-NHEJ6**	CTG--
**KI1-NHEJ7**	CTGAGTTCCTACCCCCGGCTGCGAGAGGAGACCGAGCGAATTGTCACCACCTA----TCAGGGAGCGAGAAGGGAGAACCAAGGACCAG

Supplementary Figure S1. Sequences of the NHEJ clones. Alignment of the sequences of the WT (green) and the mutated (red) mouse Dnm2 exon 11 and the NHEJ clones. Note that for KI1-NHEJ6 clone, 85 nucleotides were deleted.

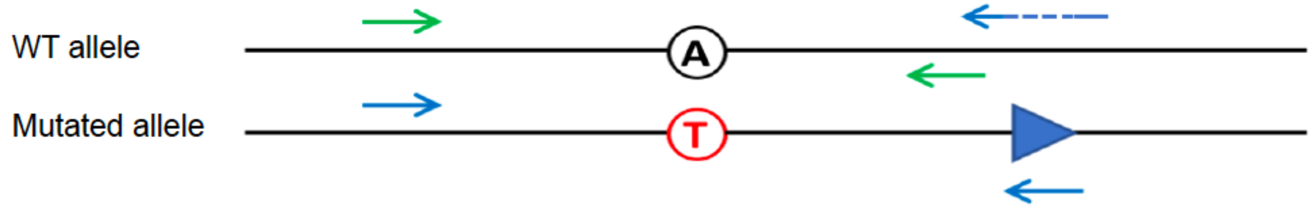

Supplementary Figure S2. Schema of *Dnm2*$^{R465W/+}$ KI mouse exon 11 and the primers used for PCR amplification. “A” represents the WT nucleotide and “T” the mutated one. The mutated allele includes a Lox-P site in the 3’-*Dnm2* exon 11 intronic region. Primers in green amplify both alleles. Primers in blue amplify specifically either the mutated or the WT allele depending on the position of the reverse primer relative to the Lox-P site (Dashed-arrow shows the WT specific reverse primer disrupted by the Lox-P site).

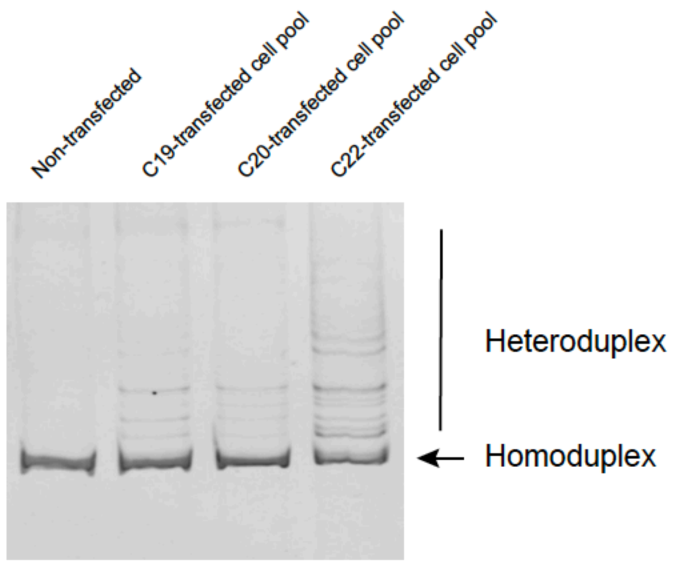

Supplementary Figure S3. Modifications of the WT allele assessed on the cell pools. *Dnm2*$^{R465W/+}$ KI myoblasts were transfected with the indicated sgRNAs and GFP sorted C19 and C20 are alle-specific sgRNAs, C22 is pan-allelic sgRNA). The WT allele was specifically amplified by PCR and after denaturation-annealing cycles, heteroduplex were formed and migrated slower than homoduplex in native PAGE 10% gel. The intensity of the bands was quantified by ImageJ software.
