# Supplementary material for: Allele-Specific CRISPR/Cas9 Correction of a Heterozygous DNM2 Mutation Rescues Centronuclear Myopathy Cell Phenotypes
Source: Mol Ther Nucleic Acids. 2019 Feb 27;16:246–56. doi: 10.1016/j.omtn.2019.02.019 (PMC6439232; doi:10.1016/j.omtn.2019.02.019)
Supplement: Document S2. Article plus supplemental information [file mmc2.pdf]

Molecular Therapy
Nucleic Acids
Original Article

AMERICAN SOCIETY of GENE & CELL THERAPY

# Allele-Specific CRISPR/Cas9 Correction of a Heterozygous *DNM2* Mutation Rescues Centronuclear Myopathy Cell Phenotypes

Aymen Rabai,[1,2,3,4] Léa Reisser,[1,2,3,4] Bernardo Reina-San-Martin,[1,2,3,4] Kamel Mamchaoui,[5] Belinda S. Cowling,[1,2,3,4] Anne-Sophie Nicot,[1,2,3,4] and Jocelyn Laporte[1,2,3,4]

[1]Institut de Génétique et de Biologie Moléculaire et Cellulaire (IGBMC), Illkirch 67404, France; [2]INSERM U1258, Illkirch 67404, France; [3]CNRS UMR7104, Illkirch 67404, France; [4]Strasbourg University, Illkirch 67404, France; [5]UMR S787, Institut de Myologie, Université Pierre et Marie Curie, 75013 Paris, France

**Genome editing with the CRISPR/Cas9 technology has emerged recently as a potential strategy for therapy in genetic diseases. For dominant mutations linked to gain-of-function effects, allele-specific correction may be the most suitable approach. In this study, we tested allele-specific inactivation or correction of a heterozygous mutation in the Dynamin 2 (*DNM2*) gene that causes the autosomal dominant form of centronuclear myopathies (CNMs), a rare muscle disorder belonging to the large group of congenital myopathies. Truncated single-guide RNAs targeting specifically the mutated allele were tested on cells derived from a mouse model and patients. The mutated allele was successfully targeted in patient fibroblasts and *Dnm2*$^{R465W/+}$ mouse myoblasts, and clones were obtained with precise genome correction or inactivation. *Dnm2*$^{R465W/+}$ myoblasts showed an alteration in transferrin uptake and autophagy. Specific inactivation or correction of the mutated allele rescued these phenotypes. These findings illustrate the potential of CRISPR/Cas9 to target and correct in an allele-specific manner heterozygous point mutations leading to a gain-of-function effect, and to rescue autosomal dominant CNM-related phenotypes. This strategy may be suitable for a large number of diseases caused by germline or somatic mutations resulting in a gain-of-function mechanism.**

## INTRODUCTION

Autosomal dominant diseases represent a challenge for development of effective therapies. Whereas haploinsufficiency may be treated by conventional gene replacement, diseases linked to gain-of-function or toxic effect in essential proteins require the targeting of the mutated allele. The mutated allele can be either inactivated, leading to haploinsufficiency, or corrected to revert to a wild-type (WT) genotype. In the case of disease-implicated genes essential for cellular functions, allele-specific correction would be preferred to avoid deleterious effects due to an overall decrease of the protein expression. In this study, we tested the potential of genome editing to rescue the phenotypes of a dominant disease through allele-specific inactivation or correction.

Allele-specific gene inactivation can be achieved through gene silencing with shRNA[1] or antisense oligonucleotides, leading to haploinsufficiency. Mutation correction can be obtained at the RNA level through *trans* splicing,[2,3] although allele-specific targeting is still challenging with this strategy. The most upstream approach would be to target the mutation at the DNA level. Genome editing using programmable nucleases has emerged as a powerful tool for targeting specific sequences. The clustered regularly interspaced short palindromic repeats (CRISPR)-related CRISPR-associated protein 9 (Cas9) system is the most commonly used tool. Cas9 endonuclease is guided to a specific DNA sequence by a single-guide RNA (sgRNA)[4] and makes a double-strand break (DSB) that triggers the cellular repair machinery. The main repair pathways are "error-prone" non-homologous end-joining (NHEJ), mainly leading to gene inactivation or the precise correction via homology-directed repair (HDR) with the help of a DNA repair template.[5] Monteys et al.[6] assessed the allele specificity of CRISPR/Cas9 based on SNPs in *cis* that form a protospacer adjacent motif (PAM) on the huntingtin mutated allele in patients' fibroblasts, and Yamamoto et al.[7] assessed the specificity of CRISPR/Cas9 to target a heterozygous single point mutation in the *CALM2* gene in human induced pluripotent stem cells. Both studies disrupted specifically the mutated allele by NHEJ but did not report allele-specific correction. Allele-specific correction in a dominant disease was achieved after the integration of an antibiotic resistance cassette to promote the selection of the corrected allele.[8]

As a paradigm for testing allele-specific genome editing without integration of a selection cassette that might affect the genome structure or regulation, we focused on a dominant form of centronuclear myopathy (CNM). CNMs are rare muscle disorders belonging to the group of congenital myopathies. Several forms of the disease have been described with different severities.[9] The autosomal dominant form is characterized by neonatal to adult onset, muscle weakness, and delayed motor milestones. Autosomal dominant CNM is caused by heterozygous mutations in the *DNM2* gene, which encodes

Received 15 November 2018; accepted 20 February 2019;
https://doi.org/10.1016/j.omtn.2019.02.019.

**Correspondence:** Jocelyn Laporte, IGBMC, 1 Rue Laurent Fries, BP10142, 67404 Illkirch, France.
**E-mail:** jocelyn@igbmc.fr

 © 2019 The Author(s).
This is an open access article under the CC BY license (http://creativecommons.org/licenses/by/4.0/).

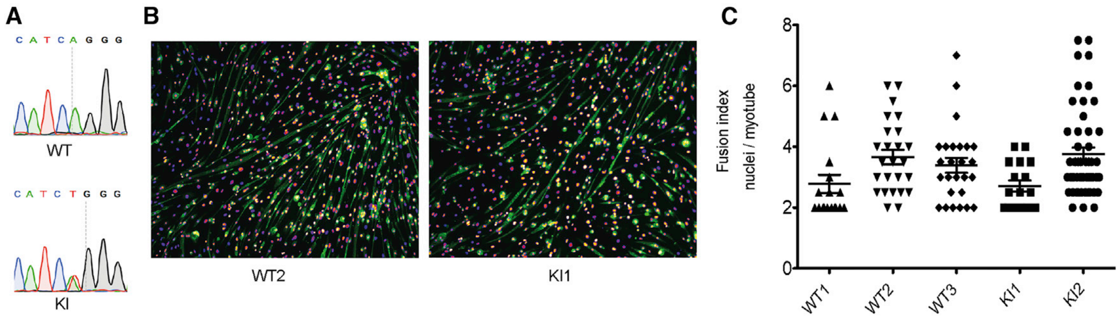


**Figure 1. Establishment of *Dnm2*$^{R465W/+}$ Knock-in Myoblasts with Myogenic Potential**

(A) Examples of Sanger sequencing for *Dnm2*$^{R465W/+}$ knock-in (KI) and wild-type immortalized myoblasts. (B) After 7 days of differentiation, immortalized *Dnm2*$^{R465W/+}$ KI and WT muscle cells were labeled with DAPI for nuclei (fire) and caveolin 3 antibody (green) for specific labeling of myotubes. (C) The fusion index, i.e., the number of nuclei per myotubes, after 7 days of differentiation, in two *Dnm2*$^{R465W/+}$ KI clones and three WT clones in duplicate. Twenty-two to 48 myotubes were assessed in duplicate. Data are expressed as the mean ± SEM. The non-parametric Kruskal-Wallis and post hoc Dunn's multiple-comparison tests showed no statistically significant differences.

for the Dynamin 2 (DNM2) GTPase enzyme.[10,11] The *DNM2* R465W point mutation represents the most frequent mutation occurring in approximately one of four patients.[12] A knock-in (KI) mouse model of this mutation has been generated and develops a progressive muscle weakness with reduced muscle force and histological features, including reduced fiber size and central accumulation of oxidative staining.[13] *In vitro*, *DNM2*-CNM mutations enhance the GTPase activity and promote oligomerization, suggesting a gain-of-function pathomechanism.[14,15] However, this gain-of-function hypothesis has not been fully validated at the cellular level. DNM2 is a ubiquitous GTPase involved in the fission of endocytic vesicles during clathrin-mediated endocytosis,[16] cytoskeleton interaction,[17–19] and autophagy regulation.[20] Alterations of transferrin or EGFR uptake in cells expressing different DNM2 mutants have been reported, suggesting a defect in endocytosis.[21–23] In mouse embryonic fibroblasts (MEFs) harboring the DNM2 R465W mutation in the homozygous state, autophagy defects were also reported.[24] However, the cellular pathomechanism of the disease is still not well understood. To date, there is no specific treatment for the autosomal dominant form of CNM, and the potential of using CRISPR/Cas9 to treat dominant inherited diseases has yet to be better explored.

The aim of this study was to assess the impact of the most common DNM2 R465W mutation on cellular pathways in myoblasts, providing a cellular context for investigating the disease, and to determine if genome editing can inactivate (NHEJ) or correct (HDR) the *DNM2* mutation in an allele-specific manner and reverse the disease-related phenotypes.

## RESULTS

### Establishment of *Dnm2*$^{R465W/+}$ KI Immortalized Myoblasts

To be able to assess the cellular pathology and the efficiency of the CRISPR/Cas9 system in cells relevant for the disease, we established *Dnm2*$^{R465W/+}$ immortalized myoblasts. Primary myoblasts were isolated from lower limb muscles of postnatal day 5 (P5) WT and *Dnm2*$^{R465W/+}$ mice and transduced with a lentivirus expressing CDK4. After antibiotic selection of CDK4-expressing clones, cell sorting, and clone expansion, three WT and two *Dnm2*$^{R465W/+}$ (thereafter named KI) clones were established. The genotype was confirmed by PCR and Sanger sequencing (Figure 1A). We verified that immortalization did not affect their ability to fuse into myotubes (Figures 1B and 1C). After 7 days in differentiation medium, all clones differentiated into myotubes, as assessed by the fusion index and the expression of caveolin 3 (Figures 1B and 1C). The DNM2 mutation had no significant impact on the DNM2 protein level in these muscle cells (Figures 2F and 2G).

### Allele-Specific Inactivation and Correction of the *DNM2* Mutation

DNM2-related CNM is mainly caused by heterozygous single point mutations. The CGG codon in humans and the AGG codon in mice (Figure 2A) code for a conserved arginine residue at amino acid position 465. In patients and *Dnm2*$^{R465W/+}$ KI mouse CGG and AGG codons are changed into TGG, encoding a tryptophan. To inactivate or correct specifically the mutated human and mouse alleles, we designed pan-allelic guide RNAs (gRNAs) targeting both the mutated and WT alleles or allele-specific gRNAs targeting only the mutated allele (Figures 2B and 2C). For the pan-allelic gRNAs, the PAM was selected precisely at the codon encoding R465 (**C**GG or **A**GG in humans and mice, respectively) or at the R465W (**T**GG) mutation. As the first nucleotide of the PAM is variable (NGG), these gRNAs should recognize both alleles. To avoid off-target effects and to provide allele-specific recognition of the mutated allele, we used 18 bp truncated gRNAs.[25] The PAM for the allele-specific gRNAs was selected downstream of the R465W (**T**GG) mutation, thus generating a 1 bp mismatch between the gRNA and the WT allele target sequence (Figures 2B and 2C). As truncated gRNAs (tru-gRNAs) are sensitive to mismatches, this method should provide allele specificity.[25]

Cells were transfected with a vector co-expressing Cas9-EGFP and the gRNA, together with a 200 bp single-stranded oligonucleotide (ssODN), HDR template bearing silent mutations allowing us to determine correct integration by restriction digest (Figures 2B

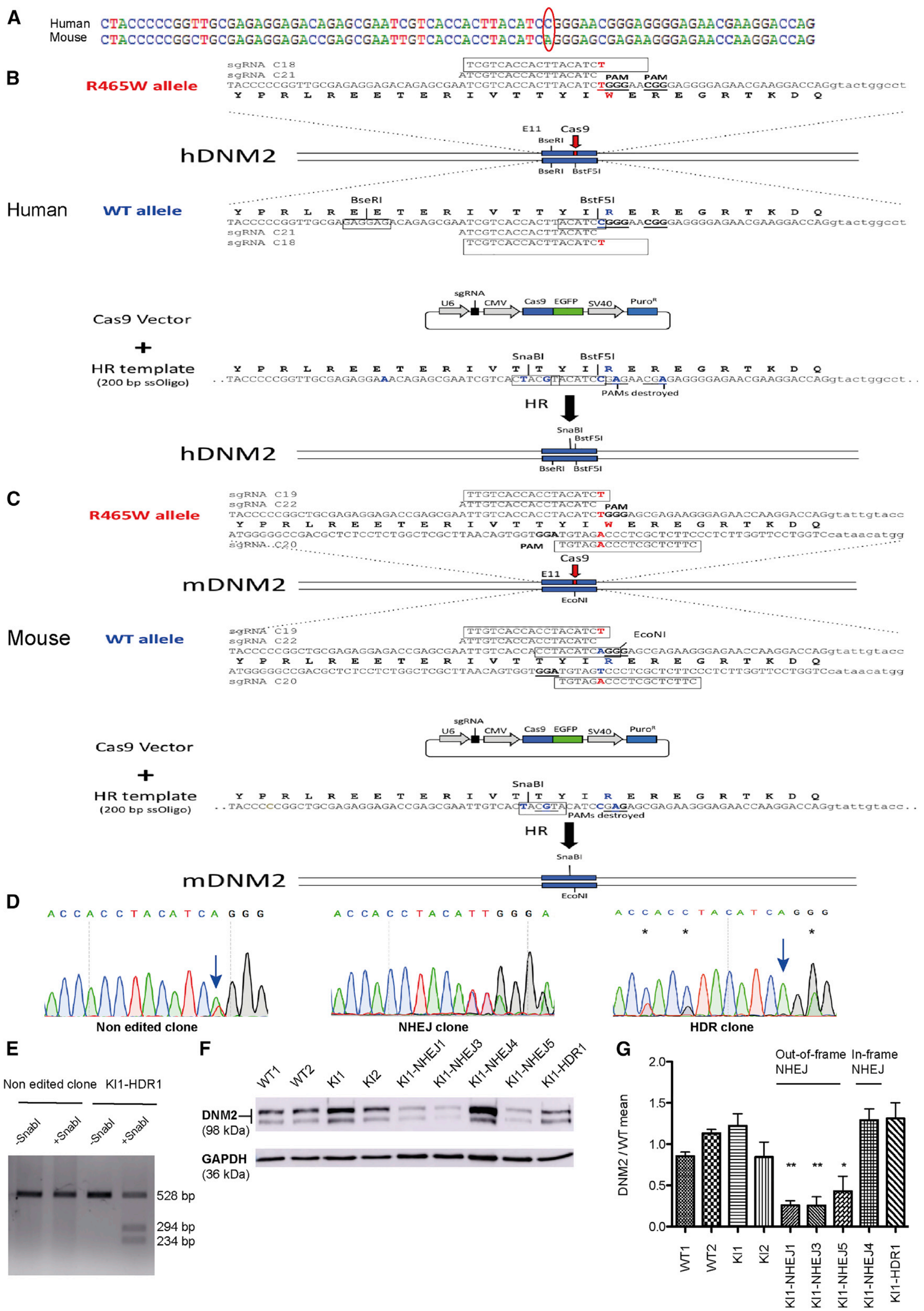


*(legend on next page)*

and 2C). Cells were sorted for EGFP expression and cultured under clonal conditions. Individual clones were isolated and genotyped by PCR, restriction digest, and Sanger sequencing, to score for inactivation of mutations through NHEJ or for correction through HDR. NHEJ-mediated repair led to small insertions or deletions (Figure 2D), with 86% of out-of-frame modifications, potentially leading to premature stop codons. Although HDR was less frequent, it allowed the correction of the *DNM2* point mutation (Figure 2D). Correct HDR events were verified by restriction digest (Figure 2E). In conclusion, both inactivation and specific correction of the mutated allele were achieved.

To test CRISPR/Cas9-mediated genome editing in human cells, we transfected HeLa cells with a pan-allelic sgRNA (HeLa cells harbor only the WT allele) and an HDR template. Genomic modification through NHEJ and HDR was obtained with an efficiency of 13.5% and 9.5%, respectively (Table 1). To validate allele-specific targeting in human cells, we used immortalized fibroblasts obtained from patients harboring the heterozygous DNM2 R465W mutation and an allele-specific gRNA. Analysis of single-cell clones showed 60% NHEJ (Table 1). Importantly, all of the NHEJ events detected occurred exclusively on the mutated allele (Table 1).

To determine whether allele-specific inactivation or correction of the DNM2 mutation can be achieved in a muscular context, two allele-specific gRNAs and one pan-allelic gRNA were transfected, together with an HDR template, into immortalized KI myoblasts derived from the *Dnm2*$^{R465W/+}$ mouse model. One of the allele-specific gRNAs led exclusively to clones with NHEJ (95%), and the other led to NHEJ (90.62%) and HDR (6.25%) (Table 1; Figure S1). As expected, in the corrected clones, the mutated T reverted to A, and three silent mutations were introduced (Figure 2D). HDR was confirmed by restriction digest (Figure 2E). Notably, whereas the pan-allelic sgRNA targeted both alleles (25% of clones modified), only the mutated allele was targeted with the allele-specific gRNAs.

In addition, allele specificity was verified by quantification, using a heteroduplex mobility assay on the cell pools. The WT allele of the C19-, C20-, and C22-transfected and GFP-sorted cell pools was specifically amplified, with a PCR primer that is disrupted by the loxP site present on the mutated allele of the KI myoblasts (Figure S2). After the denaturation-annealing cycles, heteroduplexes were formed and migrated slower than homoduplexes in native 10% PAGE, because of the open angle caused by the mismatched DNA of the indel mutations. The pan-allelic sgRNA (C22) targeted the WT allele with an efficiency of 37%, and the allele-specific sgRNAs modifications of the WT allele were 8% and 4%, respectively for the C19 and C20 sgRNAs (Figure S3). These minor modifications on the WT allele with the allele-specific sgRNAs were not detected in the clonal analysis, probably because of the lower number of analyzed events compared to the cell pools.

We assessed the effect of NHEJ modification on the DNM2 protein level. Among the isolated myoblast clones, some carried allele-specific, out-of-frame insertions-deletions (indels) and were expected to produce premature stop codons, and others carried allele-specific in-frame indels (Figure S1). The DNM2 protein level was quantified by western blot in these two types of clones (Figures 2F and 2G). In cells with out-of-frame indels (KI1-NHEJ1, KI1-NHEJ3, and KI1-NHEJ5), DNM2 was decreased to about half the level found in WT cells and in non-edited KI cells (KI1-NHEJ1: 0.27 ± 0.05; KI1-NHEJ3: 0.25 ± 0.1; and KI1-NHEJ5: 0.43 ± 0.18). However, no protein reduction was observed after allele-specific in-frame deletions (clone KI1-NHEJ4). We conclude that out-of-frame indels lead to protein disruption, supporting the theory that NHEJ modification can be used to remove the disease-causing mutated protein. Conversely, allele-specific correction by HDR leads to expression of a normal level of DNM2 (Figures 2F and 2G).

We conclude that allele-specific inactivation and/or correction of the mutated R465W allele was achieved in different cell types of both murine and human origin.

### Allele-Specific Inactivation or Correction of the DNM2 Mutation Rescue of the Alterations in Endocytosis

DNM2 is important for the fission of vesicles during clathrin-mediated endocytosis.[26] Contradictory data have been reported on the impact of DNM2-CNM mutations on endocytosis with either no effect or decreased endocytosis.[21,23,27] These data were obtained on transfected cells or on non-muscle cells. To assess the impact of the R465W mutation on the endocytic pathway in a muscular context expressing DNM2 endogenously, cyanine 3 (CY3)-labeled transferrin uptake was measured in mouse immortalized myoblasts by using

**Figure 2. Strategy and Results for Allele-Specific Inactivation and/or Correction in Muscle Cells**

(A) Alignment of the human and mouse WT sequences. The CGG codon in human (top) or the AGG codon in mouse (bottom) code for the conserved arginine residue. In human and *Dnm2*$^{R465W/+}$ KI mouse, CGG and AGG codons are changed into TGG encoding a tryptophan. Sequences of the human (B), *Dnm2*$^{R465W/+}$ KI mouse (C) mutated alleles, sgRNAs, and repair templates. Note the presence of the NGG protospacer adjacent motif (PAM) near the mutation. We designed allele-specific high-score sgRNAs and pan-allelic sgRNA. (D) Examples of chromatopherograms for a non-edited clone, an NHEJ clone, and a corrected HDR clone from *Dnm2*$^{R465W/+}$ KI mouse myoblasts. Positions of the mutated or corrected nucleotides are indicated by arrows and the silent variations introduced by HDR with the repair template are marked by stars. (E) *Sna*BI digestion of DNA from non-edited and corrected *Dnm2*$^{R465W/+}$ KI mouse myoblasts. (F) Dynamin 2 protein levels assessed by western blot from WT (WT1, WT2) and *Dnm2*$^{R465W/+}$ KI (KI1, KI2) mouse myoblasts and from *Dnm2*$^{R465W/+}$ KI myoblasts edited through out-of-frame or in-frame NHEJ or corrected through HDR. (G) Quantification from three independent experiments of DNM2 protein levels, related to GAPDH from WT (WT1, WT2) and *Dnm2*$^{R465W/+}$ KI (KI1, KI2) mouse myoblasts and from *Dnm2*$^{R465W/+}$ KI myoblasts edited through out-of-frame or in-frame NHEJ or corrected through HDR. Data are expressed as the mean ± SEM. No statistically significant difference was noted between WT and KI myoblasts by one-way ANOVA followed by Dunnett's multiple-comparison test. Repeated-measures ANOVA followed by Dunnett's multiple-comparison test between KI1 clone and KI1-derived edited clones showed that out-of-frame NHEJ led to a decreased DNM2 level, whereas the in-frame NHEJ clone and the HDR-corrected clone had a normal DNM2 level. *$p < 0.05$, **$p < 0.01$.

**Table 1. Allele-Specific Inactivation and/or Correction in Different Cell Types from Humans and Mice**

| Cell Lines | sgRNA | % NHEJ | % Allele-Specific Inactivation following NHEJ | % HDR | % Allele-Specific Correction following HDR |
|---|---|---|---|---|---|
| HeLa | C21 pan allelic | 13.5 | NA | 9.3 | NA |
| Patient fibroblasts (R465W mutation) | C18 allele specific | 60 | 100 | 0 | NA |
| *Dnm2*$^{R465W/+}$ mouse myoblasts | C19 allele specific | 95 | 100 | 0 | NA |
| | C20 allele specific | 90.6 | 100 | 6.2 | 100 |
| | C22 pan allelic | 25 | NA | 0 | NA |

More than 55 cell clones were sequenced per sgRNA that were either pan allelic or allele specific. NA, not applicable, as there was no *DNM2* mutation in HeLa cells or utilization of pan-allelic sgRNAs.

fluorescence-activated cell sorting (FACS) at the indicated times (Figure 3A). The two independent *Dnm2*$^{R465W/+}$ KI myoblasts lines showed an increase in transferrin uptake after 10 and 20 min, compared with three independent WT myoblast lines (at 10 min, KI1: 2.42 ± 0.28-fold and KI2: 1.77 ± 0.25-fold increase, and at at 20 min, KI1: 1.93 ± 0.35-fold and KI2: 2.15 ± 0.06-fold increase). To analyze the contribution of DNM2 in this phenotype, we used a dynamin pharmacological inhibitor, Dynole 34-2[28] (Figure 3B). Transferrin uptake was inhibited in WT and KI myoblasts with a concentration of 50 μM (WT1: 0.41 ± 0.04-fold and WT2: 0.26 ± 0.16-fold; WT3: 0.3 ± 0.15-fold; KI1: 0.98 ± 0.06-fold and KI2: 0.63 ± 0.13-fold), validating that the increase of transferrin uptake seen in KI myoblasts is caused by DNM2 alteration. In addition, DNM2 inhibition by Dynole 34-2 at 50 μM normalized the increase of transferrin uptake in KI clones (Figure 3B). To confirm if this endocytosis alteration is disease-relevant, we quantified the transferrin uptake in human myoblast lines (Figures 3C and 3D). Three controls and two patient lines with different *DNM2* mutations were used. Myoblasts derived from patient biopsies harboring DNM2 R465W and R369Q mutations showed an increase in transferrin uptake compared with control cells (R465W: 15.2 ± 1.11, and R369Q: 8.66 ± 0.25 mean intensity; controls are between 3.00 and 5.00 mean intensity). This phenotype was also observed in both primary and immortalized myoblasts (Figure 3D). Taken together, these data show that DNM2-CNM mutations R465W and R369Q increase endocytosis in muscle cells and behave as gain-of-function mutations.

We further investigated the potential of allele-specific genome editing to rescue this phenotype in mouse myoblasts. Allele-specific out-of-frame NHEJ inactivation of the mutated allele decreased the transferrin uptake at 10 min, compared with the mutated cells, and partially rescued or fully normalized the endocytosis phenotype (Figure 3E). Allele-specific NHEJ in-frame deletion also normalized the phenotype. Of note, both out-of-frame and in-frame NHEJ modifications rescued the phenotype, although out-of-frame NHEJ led to a decreased DNM2 protein level, whereas the in-frame NHEJ clone had a normal DNM2 level (Figures 2F and 3E), suggesting that the in-frame NHEJ led to protein inactivation as well.

In addition, using a pan-allelic sgRNA, we generated one clone (KI1-NHEJ7; Figure 3E) in which the WT allele was disrupted through out-of-frame NHEJ, whereas the mutated allele was preserved. In that specific clone, we did not observe a statistically significant difference compared to non-edited myoblasts, suggesting that the mutated protein is more active than the WT protein.

Concerning the allele-specific correction through HDR, the two tested clones rescued the endocytosis phenotype in these myoblasts (Figure 3E).

In conclusion, allele-specific inactivation or correction rescues the alteration of endocytosis found in *Dnm2*$^{R465W/+}$ KI myoblasts.

### Allele-Specific Inactivation or Correction of the DNM2 Mutation Rescues Autophagy Defects

DNM2 was proposed to play a role in autophagy via autophagic lysosome reformation.[20] Durieux et al.[24] reported a decrease in autophagy dynamics in mouse embryonic fibroblasts with a homozygous R465W mutation. However, the impact of a heterozygous DNM2 mutation in a muscle cell was never reported.

To assess the impact of the DNM2 R465W mutation on autophagy flux in myoblasts, LC3-II level was quantified by western blot in WT and *Dnm2*$^{R465W/+}$ KI immortalized myoblasts treated or not with bafilomycin A1 (BafA1) under basal and starved conditions. BafA1 inhibits the lysosomal $Na^+/H^+$ pump and, in consequence, blocks the autophagy flux at the degradation step in the autolysosomes. Under basal conditions in untreated cells, the KI myoblasts showed an increase in lipidated LC3 (LC3-II) levels compared to WT myoblasts (WT1: 1.36 ± 0.19-fold; WT2: 1.25 ± 0.49-fold; KI1: 3.46 ± 1.04-fold; and KI2: 3.3 ± 0.5-fold) (Figures 4A and 4B). To define if this observation underlies a blockade or an increase in autophagy, BafA1-treated cells were analyzed. Under basal conditions, BafA1 treatment induced an increase of around 4-fold of LC3-II in WT myoblasts (Figures 4A and 4C). However, in KI myoblasts the effect of BafA1 was increased by only 1.65- or 1.86-fold, depending on the clones. Under the starved condition, the BafA1-induced increase in LC3-II was hampered in KI myoblasts, although the difference compared with WT myoblasts was not statistically significant (Figure 4C). Altogether, these data suggest a decrease in autophagy dynamics in KI myoblasts.

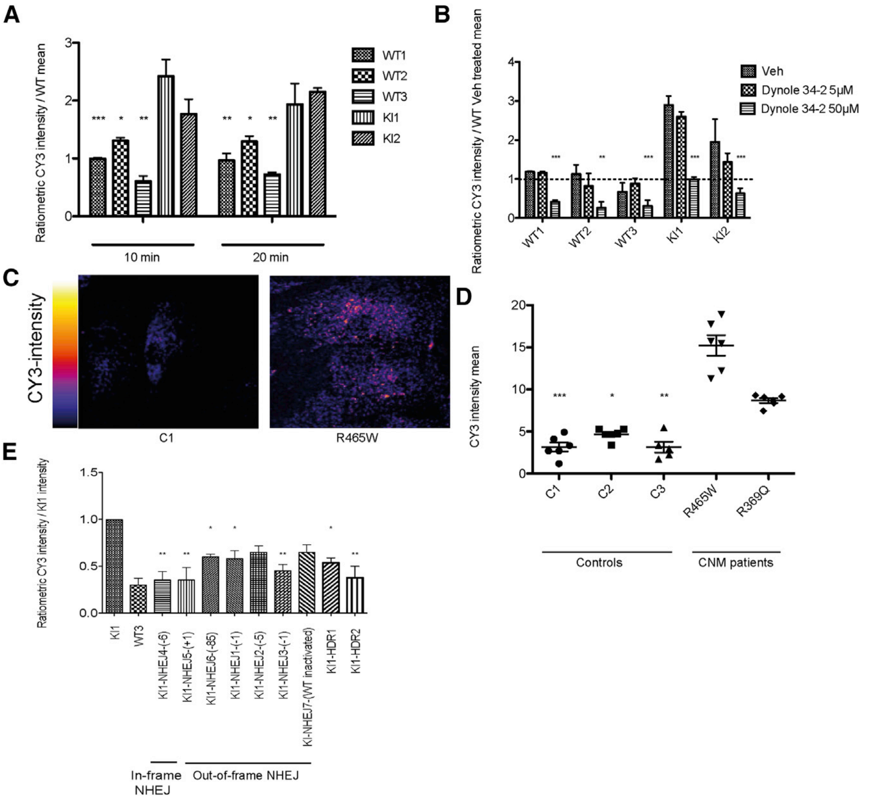


**Figure 3. Endocytosis Alteration in *Dnm2*$^{R465W/+}$ KI Myoblasts Is Rescued by Allele-Specific Inactivation or Correction**

(A) Transferrin uptake assays in three WT and two *Dnm2*$^{R465W/+}$ KI mouse myoblast lines after a 10 or 20 min incubation with CY3-labeled transferrin, normalized to the mean CY3 intensity of the WT1 cells. CY3 mean geometric fluorescence intensity was measured by FACS from three independent experiments. Data are expressed as the mean ± SEM. One-way ANOVA followed by Dunnett's multiple-comparison test. *p < 0.05, **p < 0.01, ***p < 0.001. (B) Transferrin uptake in three WT and two *Dnm2*$^{R465W/+}$ KI mouse myoblast lines after a 10 min incubation upon treatment with the dynamin inhibitor Dynole 34-2 used at two different concentrations in DMSO. CY3 mean geometric fluorescence intensity was measured by FACS from three independent experiments and normalized to the mean of WT DMSO-treated cells (represented by the dotted line). Data are expressed as the mean ± SEM. One-way ANOVA followed by Dunnett's multiple-comparison test. **p < 0.01, ***p < 0.001 versus DMSO-treated cells. (C) Primary human myoblasts from healthy or CNM patients with the R465W mutation were incubated with CY3-transferrin for 10 min and imaged by confocal microscopy. (D) Quantification of CY3 intensity of three independent experiments in duplicate, as in (C). Three WT myoblast lines and two patient myoblast lines with either an R465W or an R369Q heterozygous mutation were compared. Controls 1 and 2 and the R465W patient cells are primary myoblasts, whereas control 3 and the R369Q patient cells are immortalized myoblasts. Quantified were 125–217 cells per line. Data are expressed as the mean ± SEM. Non-parametric Kruskal-Wallis and post hoc Dunn's multiple-comparison tests. *p < 0.05, **p < 0.01, ***p < 0.001 versus R465W. (E) Quantification of three independent experiments as in (A). Transferrin uptake assays in one WT and one *Dnm2*$^{R465W/+}$ KI mouse myoblast line, seven NHEJ-derived clones, and two HDR-derived clones after 10 min incubation with CY3-labeled transferrin, normalized to the CY3 intensity of the KI1 cells. Indels are indicated in the number of nucleotides in parentheses. One in-frame NHEJ and six out-of-frame NHEJ clones were used. The KI1-NHEJ7 clone had an out-of-frame indel specifically on the WT allele. Data are expressed as the mean ± SEM. One-way ANOVA followed by Dunnett's multiple-comparison test. *p < 0.05, **p < 0.01 versus KI1. No statistically significant difference compared to WT3.

To assess whether the inactivation or the correction of the DNM2 R465W mutation could reverse the autophagy defects, the LC3-II expression level was quantified in edited myoblasts under the basal condition after BafA1 treatment. In allele-specific NHEJ inactivated clones, both out-of-frame and in-frame indels, the BafA1-induced accumulation of LC3-II was increased compared to the non-edited KI myoblasts, to the level observed in WT myoblasts (Figures 4D and 4E). In allele-specific HDR-corrected clones, we observed a similar rescue, with a BafA1-induced LC3-II:GAPDH ratio of 4.5- and 4.8-fold, depending on the clones, that was comparable to the WT value of 4.0-fold (Figures 4F and 4G). Overall, allele-specific inactivation or correction rescued the alteration of autophagy observed in the *Dnm2*$^{R465W/+}$ KI myoblasts.

## DISCUSSION

In this study, we investigated the pathomechanisms of DNM2-related CNM in muscle cells from a mouse model and patients and identified an aberrant increase in endocytosis, supporting a gain-of-function effect of DNM2 mutations. Autophagy dynamics were also altered in DNM2 mutated myoblasts. In addition, we validated allele-specific genome editing for inactivation or correction of the mutated allele for this dominant inherited disease and showed that both correlated with rescue of the different DNM2-CNM phenotypes.

### Pathomechanisms of DNM2 CNM

DNM2 is a GTPase involved in the clathrin-mediated endocytosis.[29,30] In this study, we assessed the impact of the most common heterozygous DNM2 mutation (R465W) on transferrin uptake. We observed a significant increase in transferrin uptake in *Dnm2*$^{R465W/+}$ murine myoblasts. The same phenotype was observed when CY3-labeled transferrin was visualized in patients' myoblasts compared to controls. In addition, inhibition of DNM2 activity with Dynole 34-2 suggested this phenotype is linked to dynamin. Moreover, this endocytosis increase was rescued by either inactivation or correction of the DNM2 mutation, strongly supporting that this mutation increases the rate of endocytosis.

These findings support the idea that DNM2-CNM mutations are gain-of-function, leading to a mutated protein with increased activity. This hypothesis is supported by previous *in vitro* experiments

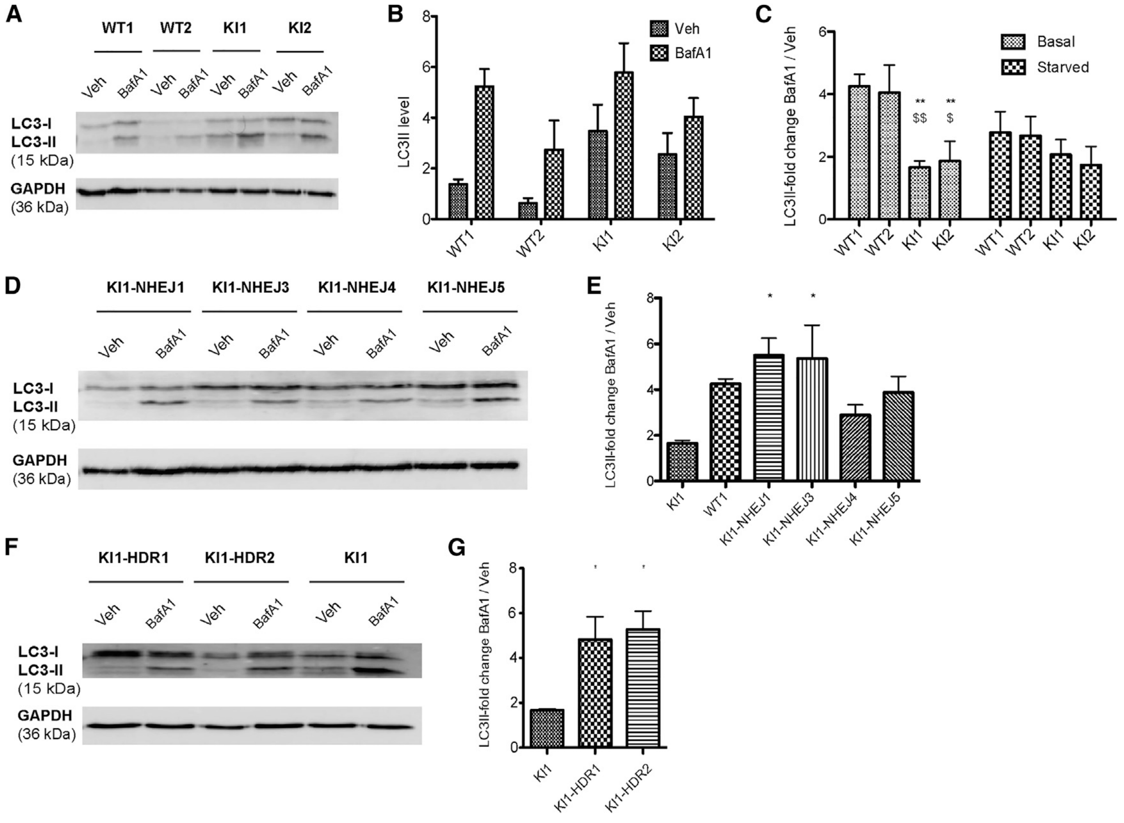


**Figure 4. Autophagy Defects in *Dnm2*$^{R465W/+}$ KI Myoblasts Are Rescued by Allele-Specific Inactivation or Correction**

(A) WT and KI immortalized myoblasts were treated or not with bafilomycin A1 (BafA1 in DMSO) under basal conditions, and lipidated LC3-II was detected by western blot. GAPDH was used as the loading control. (B) Quantification of LC3-II levels in WT and KI myoblasts under basal conditions without any treatment. Four independent experiments. Data are expressed as the mean ± SEM. One-way ANOVA followed by Bonferroni's multiple-comparison test showed no statistically significant difference between WT and KI myoblasts. (C) Quantification of BafA1-induced accumulation of LC3-II relative to GAPDH in two WT and two KI myoblast lines under basal or starved conditions, expressed as the ratio of BafA1-treated cells versus untreated cells. Four independent experiments. Data are expressed as the mean ± SEM. One-way ANOVA followed by Bonferroni's multiple-comparison test. $^{**}p < 0.01$ versus WT1; $^{\$}p < 0.05$, $^{\$\$}p < 0.01$ versus WT2. (D) Levels of LC3-II relative to GAPDH in allele-specific NHEJ inactivated myoblasts treated or not with BafA1 under basal conditions. Clone KI1-NHEJ4 has an in-frame deletion in *Dnm2*, whereas the other clones carry out-of-frame indels. GAPDH was used as the loading control. (E) Quantification of BafA1-induced accumulation of LC3-II relative to GAPDH in four allele-specific NHEJ inactivated myoblast lines under basal conditions, expressed as the ratio of BafA1-treated cells versus untreated cells. Clone KI1-NHEJ4 has an in-frame deletion in *Dnm2*, whereas the other clones carry out-of-frame indels. Three independent experiments. Ratio in the WT1 and KI1 myoblasts from (C) are shown as a comparison. Data are expressed as the mean ± SEM. One-way ANOVA followed by Bonferroni's multiple-comparison test. $^{*}p < 0.05$ versus KI1. No statistically significant difference compared with WT1. (F) Levels of LC3-II in two allele-specific HDR-corrected myoblast lines and the non-edited KI1 myoblasts, treated or not with BafA1, under basal conditions. GAPDH was used as the loading control. (G) Quantification of BafA1-induced accumulation of LC3-II relative to GAPDH in two allele-specific HDR-corrected myoblast lines and the non-edited KI1 myoblasts under basal conditions, expressed as ratio of BafA1-treated cells versus untreated cells. Three independent experiments. Data are expressed as the mean ± SEM. One-way ANOVA followed by Bonferroni's multiple-comparison test. $^{*}p < 0.05$ versus KI1.

showing that this mutation increases GTPase activity and the oligomerization of mutated DNM2 recombinant proteins.[14,15] Of note, our data showed that specific inactivation of the mutated allele (*Dnm2*$^{+/-}$ cells from out-of-frame NHEJ; Figure 3E) rescued the increased endocytosis, whereas specific inactivation of the WT allele (*Dnm2*$^{R465W/-}$ cells KI1-NHEJ7; Figure 3E) had activity similar to that of the uncorrected cells. Overall, the data support that the mutated DNM2 is more active than the WT protein.

In contrast to earlier findings, these results describe for the first time an increase in endocytosis caused by DNM2-CNM mutations. Liu et al.[23] used stable cell lines from conditional knock-out mouse fibroblasts infected with viral DNM2 vectors. They did not observe any defect in transferrin uptake related to DNM2-CNM mutations. Trochet et al.[31] demonstrated a decrease in transferrin uptake in patients' fibroblasts, whereas Koutsopoulos et al.[27] did not observe a decrease. In other experiments,[21,27] a decrease in endocytosis was observed based on transient overexpression of DNM2 mutants. We focused our work on myoblasts derived from the mouse model of the disease, to examine the Dnm2 muscle-disease-causing variant within the appropriate cellular context. In addition, these myoblasts expressed DNM2 at an endogenous level to avoid dominant negative or toxic

effects due to overexpression. We verified that the DNM2 mutation does not affect the protein level (Figure 2G).

Another phenotype that we observed in *Dnm2*$^{R465W/+}$ murine myoblasts was a defect in autophagy. These myoblasts showed an accumulation of LC3-II under the basal condition and, when autophagy flux was blocked by BafA1, LC3-II increase was compromised compared with that in WT. These findings suggest that DNM2 R465W mutation impairs autophagic degradation. Durieux et al.[24] described the impact of the DNM2 R465W homozygous mutation in mouse embryonic fibroblasts. Although their results were based on a different cell type that harbored the mutation in the homozygous stage, they described a similar defect in LC3 lipidation under BafA1 treatment. DNM2 was described as a key player in the autophagic lysosome reformation during the proto-lysosome scission.[32] In accordance, Shulze et al.[20] reported that the pharmacological inhibition of DNM2 leads to enlarged autolysosomal structures and a compromised autolysosomal degradation.

Overall, we report the alteration of several cellular pathways due to DNM2 mutations, providing novel insight into the pathophysiology mechanisms of this muscle disease. Moreover, the finding that Dynole 34-2 treatment normalizes endocytosis defects suggests that inhibiting DNM2 could be developed further for therapeutic application.

### Allele-Specific Correction of a Heterozygous Gain-of-Function Mutation

Here, we succeeded in targeting specifically the mutated allele for both allele-specific inactivation and correction in a cell model of CNM linked to heterozygous gain-of-function mutation of DNM2. To achieve allele specificity, the guide sequences encompassed the mutation site. To our knowledge, this is the first report on allele-specific CRISPR/Cas9 correction of a dominant point mutation without concomitant insertion of a resistance gene for selection. Our strategy is especially adapted for gain-of-function mutation in house-keeping genes. The potential of CRISPR/Cas9 to target specifically heterozygous single point mutations for NHEJ-mediated inactivation was reported in few studies.[6,7] Monteys et al.[6] assessed the allele specificity of CRISPR/Cas9 based on the SNPs forming a PAM in the huntingtin-mutated allele in patients' fibroblasts, whereas Yamamoto et al.[7] targeted the guide sequence to the mutation site. Further development will need to address the efficiency of allele-specific correction *in vivo*. Allele-specific inactivation of *PRKAG2* was recently achieved in heart.[33]

In addition, we used tru-gRNAs to provide allele specificity. There is still a risk of off-target effects that could be improved by the use of high-fidelity Cas9s (Cas9-HF1 or eSpCas9), which do not have detectable off-target effects and do not tolerate single base-pair mismatches between gRNA and targeted sequence.[34,35]

In the case of DNM2-CNM, we found that both the specific inactivation and the correction of the heterozygous mutation rescued alterations in endocytosis and autophagy observed in a myoblast cell model. Genome editing thus represents a novel therapeutic approach validated at the pre-clinical level for DNM2-CNM, in addition to RNA silencing[31] and acetylcholine esterase inhibitors.[36] As allele-specific correction *in vivo* may be difficult to achieve because HDR may not be active in post-mitotic muscle fibers, one would need to either correct muscle progenitors or perform allele-specific NHEJ inactivation. In the case of DNM2-CNM, the latter would be effective to potentially rescue the disease. Indeed, *Dnm2* heterozygous knock-out mice expressing 50% of DNM2 do not display obvious phenotypes,[37] and overall reduction of DNM2 through shRNA or antisense oligonucleotides does not induce detrimental effects in mice.[38,39]

In conclusion, allele-specific CRISPR/Cas9-mediated correction of heterozygous mutations represents a suitable strategy that would be applicable to a large number of diseases due to germ-line or somatic mutations.

## MATERIALS AND METHODS

### Cell Lines

Mouse primary myoblasts were extracted from hindlimbs of P5 WT and *Dnm2*$^{R465W/+}$ pups. Animal experimentation was approved by an institutional review board (APAFIS#4469 22/07/2016-21/07/2021). After collagenase (Sigma-Aldrich) and Dispase (Gibco) digestion, cell suspension was filtered through a 40 μm strainer. Non-myogenic cells were separated in a 1.5 h incubation, and myogenic cells were collected in the supernatant and plated in 1% Matrigel-coated wells in Iscove's Modified Dulbecco's Medium (IMDM; Gibco) with 20% fetal calf serum (FCS) and 1% chicken embryo extract (CEE; Gibco). Myoblasts were immortalized, using lentivirus encoding for cyclin-dependent kinase 4 (CDK4). Briefly, 100,000 cells were infected with the lentivirus overnight, using an MOI of 3, and transduced cells were selected with geneticin for 7 days. Single cells were cloned by sorting the cells by FACS (BD) into a 96-well plate. When the myoblasts reached 50% of confluence, they were transferred to a 24-well plate, then to a 6-well plate.

Patient fibroblasts harboring the DNM2 R465W mutation were immortalized by using a lentivirus encoding for the catalytic subunit of the human telomerase (hTERT) vector, as described before.[40]

### Plasmids and the Design of sgRNAs and HDR Templates

The sequence of exon 11 of the *DNM2* gene was screened for NGG proto-adjacent motif (PAM) sequences adjacent to the R(CGG) 465W(TGG) mutation in humans or to the R(AGG)465W(TGG) mutation in mice. To avoid off-target effects and to provide allele-specific recognition of the mutated allele, we used 18 bp truncated gRNAs.[25] Plasmids expressing the truncated tru-gRNA through a U6 promoter and co-expressing Cas9-EGFP through a CMV promoter were generated by Golden Gate cloning. As HDR templates, we used 200 bp ssDNA oligonucleotides (Ultramers; IDT) bearing silent mutations, to prevent Cas9-mediated cleavage of the corrected allele and to remove or add specific restriction sites for screening and verification (Figure 2B).

### Cell Culture and Transfection

HeLa cells were cultured in DMEM (Life Technologies) with 5% FCS, 100 U/mL penicillin, and 100 μg/mL streptomycin. Patient fibroblasts were cultured in DMEM with 10% FCS and 40 μg/mL gentamicin. Mouse immortalized myoblasts were cultured in IMDM (Life Technologies) supplemented with 20% FCS, 1% CEE, and 40 μg/mL gentamicin.

For transfection, when cells reached 50%–60% confluence, Sp-Cas9-EGFP and sgRNA plasmid and a ssODN as a repair template were transfected using Lipofectamine 3000 (Thermo Fisher Scientific), according to the manufacturer's protocol. GFP cells were sorted using FACS (BD) and seeded in a 6-well plate for expansion 24 h later. Two weeks later, single cells were isolated into 96-well plates by using FACS. When cells reached 50% confluence, they were transferred into a 24-well plate. Cell pellets were homogenized, and DNA of the clones was extracted using lysis buffer and proteinase K. To genotype the clones, the DNM2 region of interest was amplified, and PCR products were sequenced by Sanger sequencing. The following primers were used: human forward, 5′-CCC TGA AGC CCT GCA TGG-3′, and human reverse, 5′-GCG TGA GTT ACT ACA CCC AG-3′; and mouse forward, 5′-ACT CCA CAT GCC CCT GAC TG-3′, and mouse reverse, 5′-TGG GAG GTC GCC AGA TTA GC-3′.

### DNM2 Quantification

Mouse immortalized myoblasts were seeded into a 6-well plate, and when they reached 70% confluence, the adherent cells were mechanically scratched using RIPA buffer containing 1 mM PMSF, 1 mM DTT, and protease inhibitor cocktail (Roche Diagnostic, Basel, Switzerland) on ice. Lysates were passed through a 25G syringe and centrifuged (13,000 *g*, 4°C, 5 min). Supernatant protein concentration was determined with the BCA Protein Assay Kit 5 (Bio-Rad), using a BSA concentration range. Twenty micrograms of proteins was denatured at 95°C for 5 min. Samples were migrated on SDS-PAGE gels (10%) and transferred to nitrocellulose membranes by using the TransblotTurbo (Bio-Rad). Non-specific sites were blocked using Tris-buffered saline (TBS) containing 0.1% Tween-20 (TBST 0.1%) and 3% BSA for 1 hour. DNM2 was detected with a homemade rabbit polyclonal antibody[38] and GAPDH with a mouse monoclonal anti-GAPDH (Sigma-Aldrich) antibody in TBST 0.1% containing 0.5% BSA at 4°C overnight. Membranes were then incubated with peroxidase-conjugated secondary antibodies in TBST 0.1% and 0.5% BSA at room temperature for 45 min. Membranes were revealed with an enhanced chemiluminescence (ECL) kit (Thermo Fisher Scientific) and Imager AI 600. Protein quantification was determined with ImageJ software.

### Assessment of Myoblast Differentiation

Mouse immortalized myoblasts were cultured in Ibidi wells (Biovalley) in proliferation medium (IMDM; Life Technologies) supplemented with 20% FCS, 1% CEE, and gentamicin) until 80% confluent. Differentiation into myotubes was initiated by changing the medium to a differentiation medium containing IMDM supplemented with 3% horse serum and 40 μg/mL gentamicin. Half of the medium was changed after 3 days then every 2 days until 7 days of differentiation. Myotubes were fixed with 4% paraformaldehyde (PFA). Nuclei were stained with Hoechst, and myotubes were labeled with anti-caveolin 3 antibody (Santa Cruz) and visualized with fluorescence microscopy (Leica). The fusion index was found by counting the number of nuclei per myotube.

### Endocytosis Assays with Transferrin Uptake

For mouse immortalized myoblasts, 100,000 cells were seeded in a 24-well plate coated with 1% Matrigel. After 3 h, the cells were starved with IMDM containing 0.5% BSA for 30 min. They were then incubated with the same medium containing 20 μg/mL fluorescently labeled CY3-transferrin (Jackson ImmunoResearch Laboratories) for 10 min on ice, and endocytosis was started by incubating the cells at 37°C. At the indicated times, cells were acid washed for 3 min before fixation to remove the remaining bound transferrin at the cell surface. They were detached with trypsin, fixed with 1% PFA, resuspended in PBS, and analyzed by FACS (LSR Fortessa; BD). CY3 geometric mean intensity was measured by FACS FlowJo software. The intensity ratio of each cell line was calculated by dividing its intensity by WT mean intensity in each independent experiment.

For human myoblasts, the protocol described above was used with minor modifications. Cells (100,000) were seeded in 24-well plates containing coverslips. After 3 h, cells were starved with DMEM containing 0.5% BSA for 30 min. Cells were then incubated with the same medium containing fluorescently labeled CY3-transferrin (20 μg/mL) for 10 min on ice, and endocytosis was started by incubating the cells at 37°C. The cells were acid washed to remove the remaining bound transferrin at the cell surface, then fixed with PFA 4%. Cells were visualized using a Leica SP8 confocal microscope, and single Z-images were taken at the equatorial plane of the cells. CY3 intensity per cell was measured using ImageJ software after the subtraction of the CY3 intensity of the cells that were not incubated with CY3-transferrin.

### Autophagy Assays

Mouse immortalized myoblasts were cultured in 6-well plates in proliferation medium or starved in Hanks' balanced salt solution (HBSS; Sigma) for 2 h. In order to block the degradation of autolysosome content, the cells were treated with 15 nM BafA1 for 2 h. Adherent cells were mechanically scratched using RIPA buffer containing 1 mM PMSF, 1 mM DTT, and protease inhibitor cocktail (Roche Diagnostic) on ice. The lysates were passed through a 25G syringe and centrifuged (13,000 *g*, 4°C, 5 min). The supernatant protein concentration was determined with a BCA Protein Assay Kit 5 (Bio-Rad), using a BSA concentration range. Twenty micrograms of proteins was denatured at 95°C for 5 min. Samples were migrated on SDS-PAGE gels (15%) and transferred onto nitrocellulose membranes with the TransblotTurbo (Bio-Rad). Non-specific sites were blocked using TBST 0.1% and 3% BSA for 1 hour. LC3-I and -II were detected with a rabbit polyclonal anti-LC3 antibody (Novus) and GAPDH with a mouse monoclonal anti-GAPDH (Sigma-Aldrich) antibody

in TBST 0.1% containing 0.5% BSA at 4°C overnight. The membranes were then incubated with peroxidase-conjugated secondary antibodies in TBST 0.1% and 0.5% BSA at room temperature for 45 min. The membranes were revealed with an ECL kit (Thermo Fisher Scientific) and Imager AI 600. Protein quantification was determined with ImageJ software.

### Data Analysis and Statistics

GraphPad Prism 5.0 was used to perform statistical analysis and generate the graphs. The values are expressed as the mean ± SEM. One-way ANOVA followed by a post hoc analysis was used to compare more than two groups. Non-parametric tests were assessed when the conditions for ANOVA could not be verified (n too small, no normality). When samples were linked, repeated-measures ANOVA followed by post hoc analysis was performed.

## SUPPLEMENTAL INFORMATION

Supplemental Information can be found online at https://doi.org/10.1016/j.omtn.2019.02.019.

## AUTHOR CONTRIBUTIONS

B.S.C., A.-S.N., and J.L. designed and supervised the research. A.R. and L.R. performed the research. B.R.-S.-M. and K.M. provided materials and advice. A.R., A.-S.N., and J.L. analyzed the data. A.R. and J.L. wrote the paper.

## CONFLICTS OF INTEREST

B.S.C. and J.L. are co-founders of Dynacure, B.S.C. is now a Dynacure employee, and J.L. is a scientific advisor of Dynacure. These arrangements have been reviewed and approved by INSERM. The remaining authors declare no competing interests.

## ACKNOWLEDGMENTS

We thank Marc Bitoun for providing the *Dnm2*$^{R465W/+}$ mouse; Leigh Waddell for providing patient cells; Christine Kretz, Claudine Ebel, and Muriel Philipps for excellent technical assistance; the animal house, cell culture, flow cytometry platforms, and imaging center of IGBMC for support; and the Institut de Myologie for the human cell immortalization platform. This work was supported by INSERM, CNRS, the University of Strasbourg, the Agence Nationale de la Recherche (ANR14-CE12-0009), and grant ANR-10-LABX-0030-INRT from a French State fund managed by the ANR under the frame program Investissements d'Avenir (10-IDEX-0002). A.R. is a recipient of a doctoral fellowship from Strasbourg University.

## REFERENCES


1. Trochet, D., Prudhon, B., Vassilopoulos, S., and Bitoun, M. (2015). Therapy for dominant inherited diseases by allele-specific RNA interference: successes and pitfalls. Curr. Gene Ther. *15*, 503–510.
2. Puttaraju, M., Jamison, S.F., Mansfield, S.G., Garcia-Blanco, M.A., and Mitchell, L.G. (1999). Spliceosome-mediated RNA trans-splicing as a tool for gene therapy. Nat. Biotechnol. *17*, 246–252.
3. Trochet, D., Prudhon, B., Jollet, A., Lorain, S., and Bitoun, M. (2016). Reprogramming the Dynamin 2 mRNA by Spliceosome-mediated RNA Trans-splicing. Mol. Ther. Nucleic Acids *5*, e362.
4. Mali, P., Esvelt, K.M., and Church, G.M. (2013). Cas9 as a versatile tool for engineering biology. Nat. Methods *10*, 957–963.
5. Cox, D.B., Platt, R.J., and Zhang, F. (2015). Therapeutic genome editing: prospects and challenges. Nat. Med. *21*, 121–131.
6. Monteys, A.M., Ebanks, S.A., Keiser, M.S., and Davidson, B.L. (2017). CRISPR/Cas9 Editing of the Mutant Huntingtin Allele In Vitro and In Vivo. Mol. Ther. *25*, 12–23.
7. Yamamoto, Y., Makiyama, T., Harita, T., Sasaki, K., Wuriyanghai, Y., Hayano, M., Nishiuchi, S., Kohjitani, H., Hirose, S., Chen, J., et al. (2017). Allele-specific ablation rescues electrophysiological abnormalities in a human iPS cell model of long-QT syndrome with a CALM2 mutation. Hum. Mol. Genet. *26*, 1670–1677.
8. Smith, C., Abalde-Atristain, L., He, C., Brodsky, B.R., Braunstein, E.M., Chaudhari, P., Jang, Y.Y., Cheng, L., and Ye, Z. (2015). Efficient and allele-specific genome editing of disease loci in human iPSCs. Mol. Ther. *23*, 570–577.
9. Jungbluth, H., Wallgren-Pettersson, C., and Laporte, J. (2008). Centronuclear (myotubular) myopathy. Orphanet J. Rare Dis. *3*, 26.
10. Bitoun, M., Bevilacqua, J.A., Prudhon, B., Maugenre, S., Taratuto, A.L., Monges, S., Lubieniecki, F., Cances, C., Uro-Coste, E., Mayer, M., et al. (2007). Dynamin 2 mutations cause sporadic centronuclear myopathy with neonatal onset. Ann. Neurol. *62*, 666–670.
11. Bitoun, M., Maugenre, S., Jeannet, P.Y., Lacène, E., Ferrer, X., Laforêt, P., Martin, J.J., Laporte, J., Lochmüller, H., Beggs, A.H., et al. (2005). Mutations in dynamin 2 cause dominant centronuclear myopathy. Nat. Genet. *37*, 1207–1209.
12. Böhm, J., Biancalana, V., Dechene, E.T., Bitoun, M., Pierson, C.R., Schaefer, E., Karasoy, H., Dempsey, M.A., Klein, F., Dondaine, N., et al. (2012). Mutation spectrum in the large GTPase dynamin 2, and genotype-phenotype correlation in autosomal dominant centronuclear myopathy. Hum. Mutat. *33*, 949–959.
13. Durieux, A.C., Vignaud, A., Prudhon, B., Viou, M.T., Beuvin, M., Vassilopoulos, S., Fraysse, B., Ferry, A., Lainé, J., Romero, N.B., et al. (2010). A centronuclear myopathy-dynamin 2 mutation impairs skeletal muscle structure and function in mice. Hum. Mol. Genet. *19*, 4820–4836.
14. Kenniston, J.A., and Lemmon, M.A. (2010). Dynamin GTPase regulation is altered by PH domain mutations found in centronuclear myopathy patients. EMBO J. *29*, 3054–3067.
15. Wang, L., Barylko, B., Byers, C., Ross, J.A., Jameson, D.M., and Albanesi, J.P. (2010). Dynamin 2 mutants linked to centronuclear myopathies form abnormally stable polymers. J. Biol. Chem. *285*, 22753–22757.
16. Praefcke, G.J., and McMahon, H.T. (2004). The dynamin superfamily: universal membrane tubulation and fission molecules? Nat. Rev. Mol. Cell Biol. *5*, 133–147.
17. Ochoa, G.C., Slepnev, V.I., Neff, L., Ringstad, N., Takei, K., Daniell, L., Kim, W., Cao, H., McNiven, M., Baron, R., and De Camilli, P. (2000). A functional link between dynamin and the actin cytoskeleton at podosomes. J. Cell Biol. *150*, 377–389.
18. Gu, C., Yaddanapudi, S., Weins, A., Osborn, T., Reiser, J., Pollak, M., Hartwig, J., and Sever, S. (2010). Direct dynamin-actin interactions regulate the actin cytoskeleton. EMBO J. *29*, 3593–3606.
19. Shpetner, H.S., and Vallee, R.B. (1989). Identification of dynamin, a novel mechanochemical enzyme that mediates interactions between microtubules. Cell *59*, 421–432.
20. Schulze, R.J., Weller, S.G., Schroeder, B., Krueger, E.W., Chi, S., Casey, C.A., and McNiven, M.A. (2013). Lipid droplet breakdown requires dynamin 2 for vesiculation of autolysosomal tubules in hepatocytes. J. Cell Biol. *203*, 315–326.
21. Bitoun, M., Durieux, A.C., Prudhon, B., Bevilacqua, J.A., Herledan, A., Sakanyan, V., Urtizberea, A., Cartier, L., Romero, N.B., and Guicheney, P. (2009). Dynamin 2 mutations associated with human diseases impair clathrin-mediated receptor endocytosis. Hum. Mutat. *30*, 1419–1427.
22. Koutsopoulos, O.S., Koch, C., Tosch, V., Böhm, J., North, K.N., and Laporte, J. (2011). Mild functional differences of dynamin 2 mutations associated to centronuclear myopathy and Charcot-Marie Tooth peripheral neuropathy. PLoS ONE *6*, e27498.
23. Liu, Y.W., Lukiyanchuk, V., and Schmid, S.L. (2011). Common membrane trafficking defects of disease-associated dynamin 2 mutations. Traffic *12*, 1620–1633.
24. Durieux, A.C., Vassilopoulos, S., Lainé, J., Fraysse, B., Briñas, L., Prudhon, B., Castells, J., Freyssenet, D., Bonne, G., Guicheney, P., and Bitoun, M. (2012). A centronuclear myopathy: dynamin 2 mutation impairs autophagy in mice. Traffic *13*, 869–879.

25. Fu, Y., Sander, J.D., Reyon, D., Cascio, V.M., and Joung, J.K. (2014). Improving CRISPR-Cas nuclease specificity using truncated guide RNAs. Nat. Biotechnol. *32*, 279–284.

26. Kasai, K., Shin, H.W., Shinotsuka, C., Murakami, K., and Nakayama, K. (1999). Dynamin II is involved in endocytosis but not in the formation of transport vesicles from the trans-Golgi network. J. Biochem. *125*, 780–789.

27. Koutsopoulos, O.S., Koch, C., Tosch, V., Böhm, J., North, K.N., and Laporte, J. (2011). Mild functional differences of dynamin 2 mutations associated to centronuclear myopathy and Charcot-Marie Tooth peripheral neuropathy. PLoS ONE *6*, e27498.

28. Robertson, M.J., Deane, F.M., Robinson, P.J., and McCluskey, A. (2014). Synthesis of Dynole 34-2, Dynole 2-24 and Dyngo 4a for investigating dynamin GTPase. Nat. Protoc. *9*, 851–870.

29. Chen, M.S., Obar, R.A., Schroeder, C.C., Austin, T.W., Poodry, C.A., Wadsworth, S.C., and Vallee, R.B. (1991). Multiple forms of dynamin are encoded by shibire, a Drosophila gene involved in endocytosis. Nature *351*, 583–586.

30. Altschuler, Y., Barbas, S.M., Terlecky, L.J., Tang, K., Hardy, S., Mostov, K.E., and Schmid, S.L. (1998). Redundant and distinct functions for dynamin-1 and dynamin-2 isoforms. J. Cell Biol. *143*, 1871–1881.

31. Trochet, D., Prudhon, B., Beuvin, M., Peccate, C., Lorain, S., Julien, L., Benkhelifa-Ziyyat, S., Rabai, A., Mamchaoui, K., Ferry, A., et al. (2018). Allele-specific silencing therapy for Dynamin 2-related dominant centronuclear myopathy. EMBO Mol. Med. *10*, 239–253.

32. Chen, Y., and Yu, L. (2018). Development of Research into Autophagic Lysosome Reformation. Mol. Cells *41*, 45–49.

33. Xie, C., Zhang, Y.P., Song, L., Luo, J., Qi, W., Hu, J., Lu, D., Yang, Z., Zhang, J., Xiao, J., et al. (2016). Genome editing with CRISPR/Cas9 in postnatal mice corrects PRKAG2 cardiac syndrome. Cell Res. *26*, 1099–1111.

34. Kleinstiver, B.P., Pattanayak, V., Prew, M.S., Tsai, S.Q., Nguyen, N.T., Zheng, Z., and Joung, J.K. (2016). High-fidelity CRISPR-Cas9 nucleases with no detectable genome-wide off-target effects. Nature *529*, 490–495.

35. Slaymaker, I.M., Gao, L., Zetsche, B., Scott, D.A., Yan, W.X., and Zhang, F. (2016). Rationally engineered Cas9 nucleases with improved specificity. Science *351*, 84–88.

36. Robb, S.A., Sewry, C.A., Dowling, J.J., Feng, L., Cullup, T., Lillis, S., Abbs, S., Lees, M.M., Laporte, J., Manzur, A.Y., et al. (2011). Impaired neuromuscular transmission and response to acetylcholinesterase inhibitors in centronuclear myopathies. Neuromuscul. Disord. *21*, 379–386.

37. Cowling, B.S., Chevremont, T., Prokic, I., Kretz, C., Ferry, A., Coirault, C., Koutsopoulos, O., Laugel, V., Romero, N.B., and Laporte, J. (2014). Reducing dynamin 2 expression rescues X-linked centronuclear myopathy. J. Clin. Invest. *124*, 1350–1363.

38. Tasfaout, H., Buono, S., Guo, S., Kretz, C., Messaddeq, N., Booten, S., Greenlee, S., Monia, B.P., Cowling, B.S., and Laporte, J. (2017). Antisense oligonucleotide-mediated Dnm2 knockdown prevents and reverts myotubular myopathy in mice. Nat. Commun. *8*, 15661.

39. Tasfaout, H., Lionello, V.M., Kretz, C., Koebel, P., Messaddeq, N., Bitz, D., Laporte, J., and Cowling, B.S. (2018). Single Intramuscular Injection of AAV-shRNA Reduces DNM2 and Prevents Myotubular Myopathy in Mice. Mol. Ther. *26*, 1082–1092.

40. Auré, K., Mamchaoui, K., Frachon, P., Butler-Browne, G.S., Lombès, A., and Mouly, V. (2007). Impact on oxidative phosphorylation of immortalization with the telomerase gene. Neuromuscul. Disord. *17*, 368–375.

OMTN, Volume *16*

## Supplemental Information

# Allele-Specific CRISPR/Cas9 Correction of a Heterozygous *DNM2* Mutation Rescues Centronuclear Myopathy Cell Phenotypes

**Aymen Rabai, Léa Reisser, Bernardo Reina-San-Martin, Kamel Mamchaoui, Belinda S. Cowling, Anne-Sophie Nicot, and Jocelyn Laporte**

| | |
|---|---|
| WT allele | CTGAGTTCCTACCCCCGGCTGCGAGAGGAGACCGAGCGAATTGTCACCACCTACATCAGGGAGCGAGAAGGGAGAACCAAGGACCAG |
| Mutated allele | CTGAGTTCCTACCCCCGGCTGCGAGAGGAGACCGAGCGAATTGTCACCACCTACATCTGGGAGCGAGAAGGGAGAACCAAGGACCAG |
| KI1-NHEJ1 | CTGAGTTCCTACCCCCGGCTGCGAGAGGAGACCGAGCGAATTGTCACCACCTAC--TCTGGGAGCGAGAAGGGAGAACCAAGGACCAG |
| KI1-NHEJ2 | CTGAGTTCCTACCCCCGGCTGCGAGAGGAGACCGAGCGAATTGTCACCAC----------TCTGGGAGCGAGAAGGGAGAACCAAGGACCAG |
| KI1-NHEJ3 | CTGAGTTCCTACCCCCGGCTGCGAGAGGAGACCGAGCGAATTGTCACCACCTAC--TCTGGGAGCGAGAAGGGAGAACCAAGGACCAG |
| KI1-NHEJ4 | CTGAGTTCCTACCCCCGGCTGCGAGAGGAGACCGAGCGAATTGTCACCA------------TCTGGGAGCGAGAAGGGAGAACCAAGGACCAG |
| KI1-NHEJ5 | CTGAGTTCCTACCCCCGGCTGCGAGAGGAGACCGAGCGAATTGTCACCACCTACATTTCTGGGAGCGAGAAGGGAGAACCAAGGACCAG |
| KI1-NHEJ6 | CTG------------------------------------------------------------------------------------------------------------------------------ |
| KI1-NHEJ7 | CTGAGTTCCTACCCCCGGCTGCGAGAGGAGACCGAGCGAATTGTCACCACCTA----TCAGGGAGCGAGAAGGGAGAACCAAGGACCAG |

**Supplementary Figure S1. Sequences of the NHEJ clones**. Alignment of the sequences of the WT (green) and the mutated (red) mouse Dnm2 exon 11 and the NHEJ clones. Note that for KI1-NHEJ6 clone, 85 nucleotides were deleted.

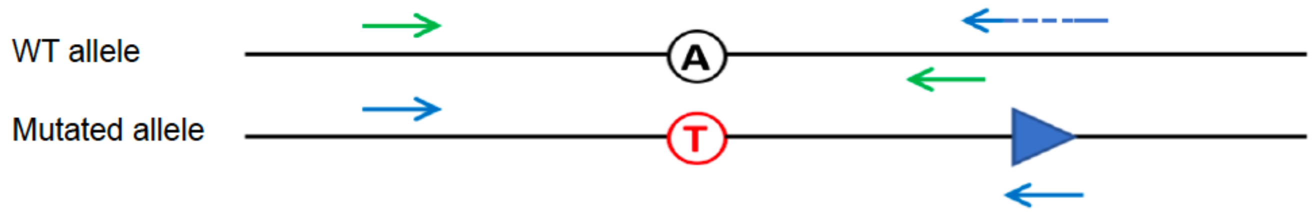


**Supplementary Figure S2. Schema of *Dnm2*$^{R465W/+}$ KI mouse exon 11 and the primers used for PCR amplification**. “A” represents the WT nucleotide and “T” the mutated one. The mutated allele includes a Lox-P site in the 3’-*Dnm2* exon 11 intronic region. Primers in green amplify both alleles. Primers in blue amplify specifically either the mutated or the WT allele depending on the position of the reverse primer relative to the Lox-P site (Dashed-arrow shows the WT specific reverse primer disrupted by the Lox-P site).

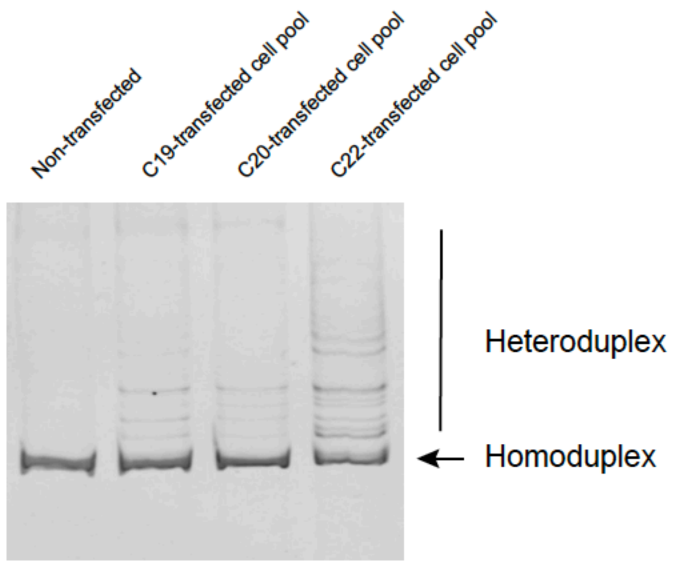


**Supplementary Figure S3. Modifications of the WT allele assessed on the cell pools**. *Dnm2*$^{R465W/+}$ KI myoblasts were transfected with the indicated sgRNAs and GFP sorted C19 and C20 are alle-specific sgRNAs, C22 is pan-allelic sgRNA). The WT allele was specifically amplified by PCR and after denaturation-annealing cycles, heteroduplex were formed and migrated slower than homoduplex in native PAGE 10% gel. The intensity of the bands was quantified by ImageJ software.
